# Evidence for chronic headaches induced by pathological changes of myodural bridge complex

**DOI:** 10.1038/s41598-024-55069-7

**Published:** 2024-03-04

**Authors:** Xue Song, Sheng-Bo Yu, Xiao-Ying Yuan, M. Adeel Alam Shah, Chan Li, Yan-Yan Chi, Nan Zheng, Hong-Jin Sui

**Affiliations:** Department of Anatomy, College of Basic Medicine, Dalian Medical University, Dalian, 116044 China

**Keywords:** Myodural bridge, Myodural bridge complex, Chronic headache, Referred pain, Bleomycin, Neuroscience, Anatomy, Medical research

## Abstract

Clinical studies have shown that there may be a certain relationship between pathological changes of the myodural bridge complex (MDBC) and chronic headaches of unknown cause. But there is still a lack of experimental evidence to explain the possible mechanism. This study aims to further confirm this relationship between MDBC and chronic headaches and explore its potential occurrence mechanism in rats. Bleomycin (BLM) or phosphate-buffered saline (PBS) was injected into the myodural bridge fibers of rats to establish the hyperplastic model of MDBC. After 4 weeks, the occurrence of headaches in rats was evaluated through behavioral scores. The immunohistochemistry staining method was applied to observe the expression levels of headache-related neurotransmitters in the brain. Masson trichrome staining results showed that the number of collagen fibers of MDBC was increased in the BLM group compared to those of the other two groups. It revealed hyperplastic changes of MDBC. The behavioral scores of the BLM group were significantly higher than those of the PBS group and the blank control group. Meanwhile, expression levels of CGRP and 5-HT in the headache-related nuclei of the brain were increased in the BLM group. The current study further confirms the view that there is a relationship between pathological changes of MDBC and chronic headaches of unknown cause. This study may provide anatomical and physiological explanations for the pathogenesis of some chronic headaches of unknown cause.

## Introduction

According to the criteria of the International Headache Society, headaches can be generally classified as primary or secondary^1^. Both types of headaches could progress into chronic headaches. A headache that occurs for more than 15 days per month and lasts at least 3 months is typically defined as a chronic headache^2^. However, the etiology of most chronic headaches is still complex and multifactorial. In recent years, clinical researchers have proposed the hypothesis that the myodural bridge (MDB)/myodural bridge complex (MDBC) may play a significant role in unexplained chronic headaches^3–5^.

The MDBC is a functional and structural unit consisting of MDB fibers, the deep suboccipital muscles (excluding obliquus capitis superior muscle), the nuchal ligament, and the vertebrodural ligament^6^. The difference between the suboccipital muscles (excluding obliquus capitis superior muscle) and other deep muscles of the upper cervical spine is that the suboccipital muscles emit MDB fibers, which are the connective tissue bridge between the spinal dura mater (SDM) and the suboccipital muscles^7,8^.

The physiological functions of MDB fibers include providing resistance to pain-sensitive SDM folding during normal head movements, transmitting cervical proprioception, and reducing stimulation of nociceptive pain mechanisms^8^. In addition, it is important that MDB may be one of the power sources for cerebrospinal fluid (CSF) circulation and a key point for some chronic headaches for unknown reasons^3,4,9,10^. The suboccipital muscles are attached to the atlanto-occipital and atlanto-axial joints, which play an important role in maintaining posture stability^11^. Besides, the suboccipital muscles have high-density muscle spindles that can move flexibly and serve as specific sensory receptors^12,13^. Several researchers have reported that malformations, defects, and abnormalities of the suboccipital muscles are associated with headaches and neck pain^14,15^. These findings suggested a possible role for the MDBC in the pathogenesis of chronic headaches.

Calcitonin gene-related peptide (CGRP) is a 37-amino acid peptide that is involved in the transmission and modulation of nociceptive signals. Increased release of CGRP is believed to promote the development of central sensitization and enhanced pain sensory^16^. As a neurotransmitter, serotonin [5-hydroxytryptamine (5-HT)] in the central nervous system exerts its core function through the descending pain pathway^17^. The 5-HT system has the dual effect of promotion and inhibition in the regulation of pain processes^17^. Elevated expression levels of CGRP or 5-HT were indicative of the occurrence of headaches. In addition, it has been reported that certain behavioral abnormalities in rats would be present during the emergence of headaches, such as cage scratching, head scratching, fro-movement, and other activities^18–20^. These behaviors of rats could objectively reflect the intensity and validity of headaches in rats, which were an important observation index of experimental headache animal models^18–20^.

However, there is still a lack of evidence regarding the relationship between pathological changes of the MDBC and chronic headaches of unknown etiology, and the potential mechanism of this relationship remains unclear. Therefore, in the present study, bleomycin (BLM) was injected into MDB fibers to establish the hyperplastic model of MDBC. The behavioral observation and immunohistochemistry staining methods were applied to observe headache-related changes in rats and further analyze the underlying mechanism. It may provide anatomical and physiological explanations for the pathogenesis of some chronic headaches of unknown etiologies.

## Result

### Hyperplastic changes of the MDBC were induced by the bleomycin injection in rats

**Figure 1:**
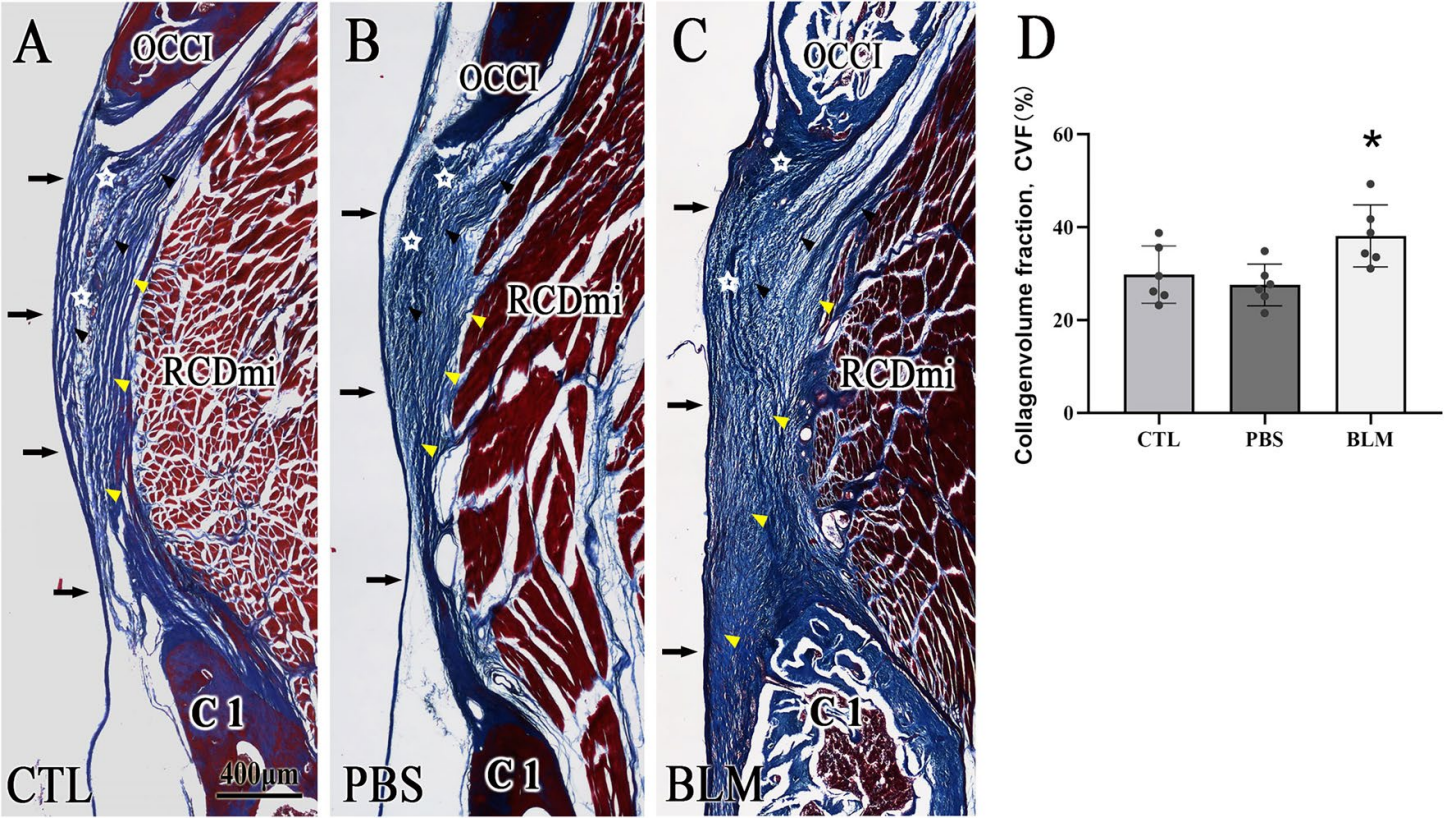
BLM-induced hyperplasia of the MDBC after a single local injection. Images display the MDBC in sagittal sections obtained from the posterior atlanto-occipital region, with Masson staining in the BLM, PBS, and CTL groups. The scale bar indicated in image (**A**) is the same as in images (**B**) and (**C**). The posterior atlanto-occipital membrane (☆), MDBC fibers (black and yellow ▲), and RCDmi appeared denser in the BLM group. In addition, the MDB fibers in the BLM group had a wider connection with the SDM, (➡). The black and yellow arrows refer to fibers of the MDBC indirectly and directly connected to the SDM respectively. Image D shows the comparison of the collagen volume fraction with Masson staining. OCCI, occipital bone; C1, atlas; RCDmi, rectus capitis dorsal minor muscle.

Masson staining revealed that the number of collagen fibers of the MDBC in the BLM group compared with the other two groups was increased in the paracentral median sagittal sections of the posterior atlanto-occipital interspace in rats. Furthermore, these fibers were arranged more closely, especially at the extensive junction between the MDBC fibers and the SDM. Meanwhile, similar changes were also observed in the posterior atlanto-occipital membrane in the BLM group (Fig. 1A–C). Semi-quantitative analysis by Image J software was performed on the Masson staining sections of rats. The results showed that the content of collagen fibers in the posterior atlanto-occipital interspace in the BLM group was significantly increased compared with the phosphate-buffered saline (PBS) group and blank control (CTL) group (P < 0.05) (Fig. 1D). It means that the amount of MDBC fibers was increased after the injection of bleomycin. These results further showed that the MDBC appeared to have hyperplastic changes, and the MDBC hyperplasia model was established successfully in rats.

### The behavioral scores of headache-related behaviors were increased in the MDBC hyperplasia rats

After 4 weeks, these headache-related behavioral motions, including cage scratching, head scratching, fro-movement, grooming episodes, and nesting, were observed for 7 days. The rats in the BLM group exhibited a higher frequency of headache-related behavioral motions. Based on statistical analysis, our results revealed that 4 weeks after bleomycin injection, the headache-related behavioral scores of rats in the BLM group were significantly higher than those in the PBS and CTL groups (P < 0.05). The behavioral scores were similar among the three groups before the bleomycin injection (P > 0.05) (Table 1). Moreover, in the BLM group, the behavioral scores were significantly higher after injection compared with those before injection (P < 0.05). No statistical difference was found before or after injections in the PBS and CTL groups (P > 0.05) (Table 1).

**Table 1:** Comparison of behavioral scores before and after drug injection.

Groups	Behavioral scores (scores/4 h)	
	Before injection	After injection
CTL	32.86 ± 4.906	30.93 ± 4.643
PBS	35.14 ± 4.465	31.55 ± 4.075
BLM	33.10 ± 2.118	44.26 ± 3.678** ^▲▲^

Comparison among three groups: **P < 0.01.

Comparison between before and after BLM injection: ^▲▲^P < 0.01.

### The expression levels of CGRP and 5-HT were significantly increased in the MDBC hyperplasia rats

**Figure 2:**
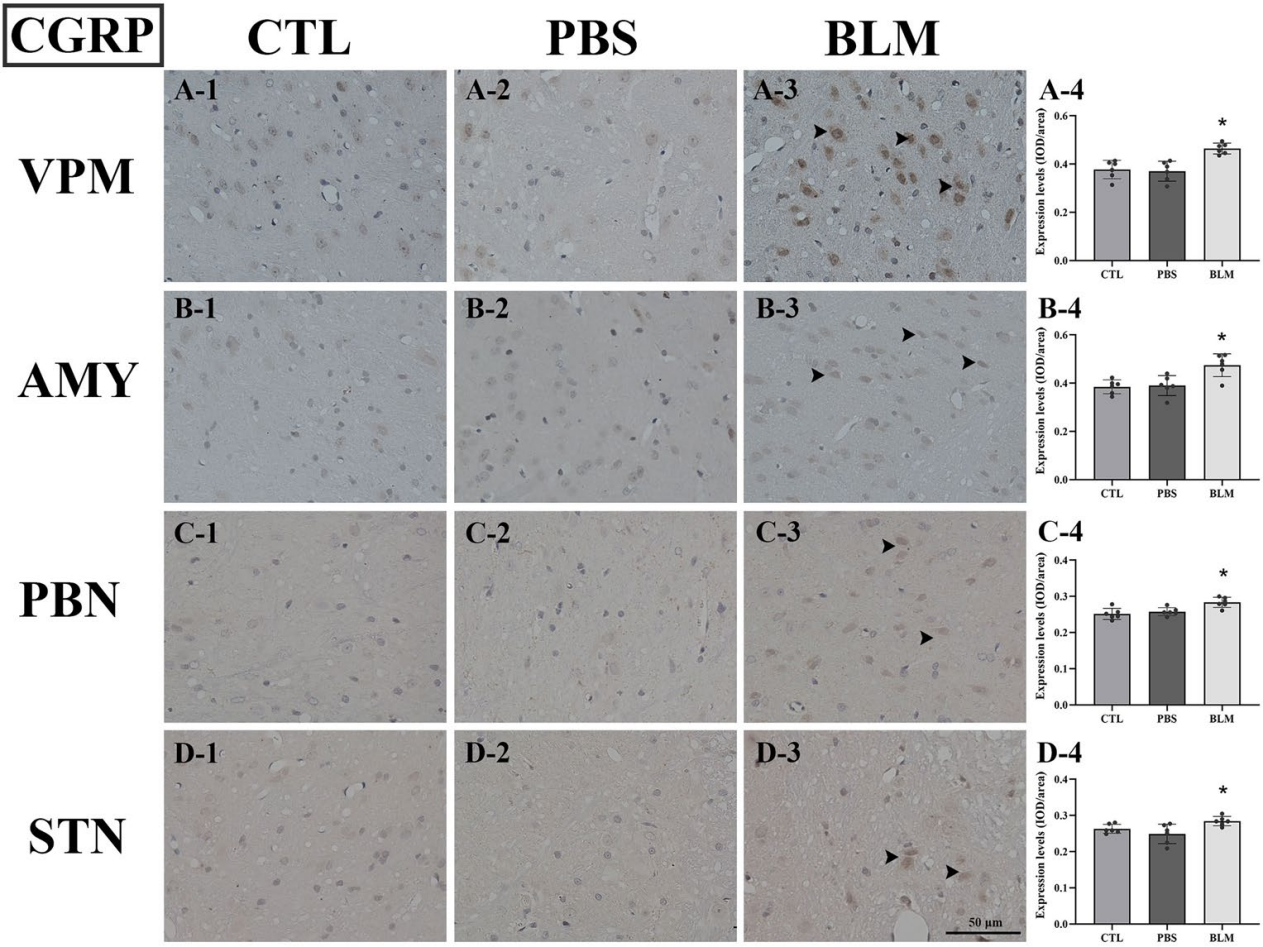
The expression levels of CGRP were significantly increased in the MDBC hyperplasia rats in the BLM, compared with the PBS and CTL groups, as shown by immunohistochemical staining. The scale bar is for all figures. VPM, ventral posteromedial nucleus of the thalamus; AMY: amygdala; PBN: parabrachial nucleus; STN: spinal trigeminal nucleus. The immunoreactive cells were stained yellow–brown, as indicated by the black arrow.

**Figure 3:**
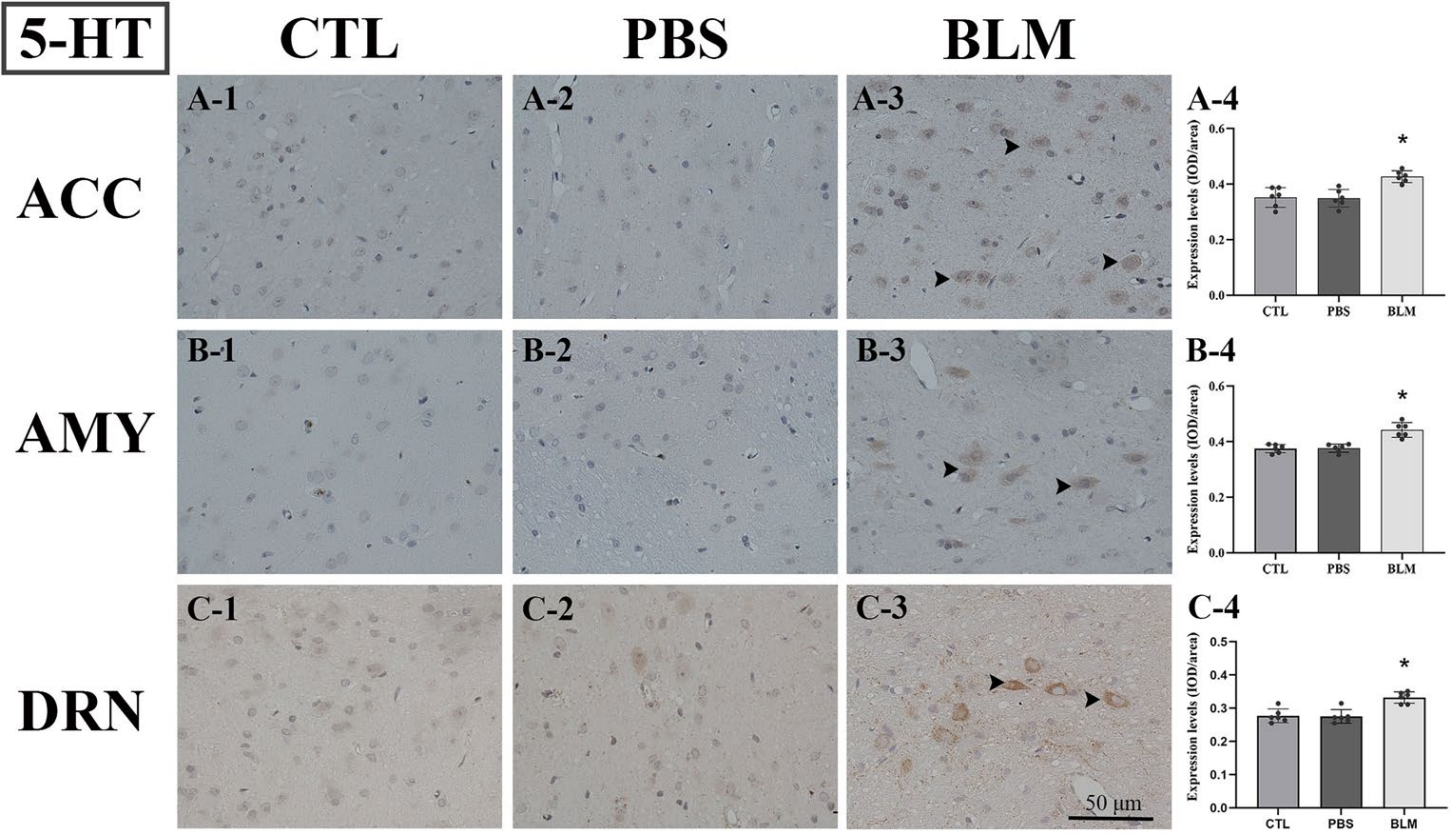
The expression levels of 5-HT were significantly increased in the MDBC hyperplasia rats in the BLM, compared with the PBS and CTL groups, as shown by immunohistochemical staining. The scale bar is for all figures. ACC, anterior cingulate cortex; AMY, amygdala; DRN, dorsal raphe nucleus. The immunoreactive cells were stained in yellow–brown, as indicated by the black arrow.

The results of immunohistochemical staining showed that the expression levels of CGRP and 5-HT were elevated in some specific nuclei of the brain tissues of rats in the BLM group. In comparison to the PBS and CTL groups, CGRP expression levels in the spinal trigeminal nucleus (STN), ventral posterior medial nucleus (VPM) of the thalamus, amygdala (AMY), and parabrachial nucleus (PBN) were higher in the BLM group (P < 0.05) (Fig. 2). And the expression levels of 5-HT in the AMY, anterior cingulate cortex (ACC), and dorsal raphe nucleus (DRN) were also increased in the BLM group compared to those of the other groups (P < 0.05) (Fig. 3).

## Discussion

In this study, we found that headache-related behaviors increased after hyperplastic changes of the MDBC were induced by the injection of bleomycin in rats. Meanwhile, expression levels of CGRP and 5-HT were both elevated in the nuclei of the brain. These findings indicated that hyperplastic changes of the MDBC could be accompanied by the occurrence of headaches. That means that there is a certain relationship between hyperplastic changes of the MDBC and chronic headaches.

In our previous animal study, according to the fibrogenic effect of bleomycin, we found that injection of bleomycin into the posterior atlanto-occipital interspace of rats led to proliferative changes in the MDBC fibers and an increased sectional area of the RCDmi, as demonstrated by histological and semi-quantitative studies^21^. Thus, the same methods were used in the present study and yielded consistent results. The contraction of the rectus capitis posterior minor muscle (RCPmi), rectus capitis posterior major muscle (RCPma) and obliquus capitis inferior muscle (OCI) during head movement leads to the tension of MDB fibers and transmits force through them to the SDM^6,8^. In other words, mechanical traction of the MDBC can cause movement of the SDM at the cranio-cervical junction. However, hyperplastic changes of MDBC might cause a failure to maintain and transmit constant tension. It would lead to a series of abnormal clinical manifestations.

It has been reported in our previous study that hyperplastic changes of the MDBC in this model resulted in increased stiffness of the SDM and reduced compliance of various structures within the cranio-cervical junction^21^. In a hypothesis study about the pathophysiology of symptomatic adult Chiari malformation Type I, it was mentioned that the stiff dura may limit the natural expansion of the subarachnoid space, thereby changing the pressure environment of CSF flow^22^. These previous studies helped us understand these pathological changes of MDBC hyperplasia which could extend to the SDM connected with it. These hyperplastic changes could reduce the compliance of various tissues in the atlanto-occipital interspace, which would weaken the promotion effect on CSF flow in this region.

The MDBC plays important functions, particularly serving as a significant contributor to CSF circulation^6,9,10,23–26^. The contraction of suboccipital musculature and ligaments could generate mechanical forces, which are exerted on the SDM through the transmission of the MDBC fibers^27,28^. This might lead to changes in the volume of the subarachnoid space in the upper cervical part, forming local negative pressure^6,10,23^. This effect, which works as a pump, would contribute to the circulation of CSF in the spinal canal^9,27^. It has been suggested that MDBC could provide an important part of the force for CSF circulation.

It had been well documented that the pathological changes of the MDBC might be relevant to the onset of chronic headaches^3,4^. But its pathogenesis remained poorly understood. Hack et al.^3^ reported a patient whose chronic headache was relieved after surgical excision of the MDB from the suboccipital musculature. They speculated that in some cases, headaches might be caused by increased tension in the suboccipital musculature, which was transmitted to the pain-sensitive SDM through the MDB fibers^3^. Yuan et al.^4^ compared cross-sectional areas of RCPmi in magnetic resonance imaging between patients with chronic headaches and healthy adult volunteers. It was found that chronic headaches were correlated with hypertrophy of the RCPmi. They presumed that RCPmi hypertrophy might affect the normal circulation of CSF, resulting in chronic headaches^4^. Moreover, it is well known that headache is one of the most common symptoms of intracranial pressure changes caused by abnormal CSF circulation^29^. A recent study indicated that the decrease of both CSF flow and velocity in headache patients might reduce the buffering capacity of the subarachnoid space as a buffer system^30^. The stimulation produced by its poor buffering capacity exceeded the threshold value of the intracranial hyperalgesia nerve, thus inducing headache^30^. Therefore, there is a high possibility of some unknown relationship between the MDBC, CSF circulation, and chronic headaches.

In this experiment, the behavioral scores of rats with MDBC hyperplasia were higher compared to those of the other two groups. In addition, significant statistical differences were also observed in the behavioral scores of the MDBC hyperplasia rats before and after bleomycin injection. Therefore, these behavioral findings preliminarily indicated that the MDBC hyperplastic rats have headache-related symptoms. In addition, increased expression levels of CGRP or 5-HT were observed by immunohistochemical staining in some specialized nuclei of the MDBC hyperplasia rats. Many studies indicated that CGRP and 5-HT played important roles in headaches^31^. These findings further supported the relationship between hyperplastic changes of the MDBC and chronic headaches.

The dorsal raphe nucleus (DRN), which contains 5-HT neurons distributed widely, is involved in the inhibition of pain through engagement with the descending pain modulation system^32,33^. Our findings showed that the expression levels of 5-HT in the DRN were increased in the BLM group compared to those of the other groups. It means that the descending pain inhibitory system is likely to be activated. Then, the 5-HT neurons from the DRN were projected to the AMY, which could promote anxiety in response to aversive stimuli^31^. Moreover, the ACC is an important cortical region for chronic pain and is related to emotional disorders^34^. The ACC receives serotoninergic neural projections originating from the DRN^34^. Simultaneously, the ACC and AMY have neural connections between each other, which are importantly implicated in the processing of mood regulation, such as fear and anxiety^35^. As an essential regulatory region of pain, the PBN was the most prominent site of CGRP expression in the brain^36,37^. CGRP-expressing neurons in the PBN could be projected to the nociceptive neurons of AMY and participate in aversion learning in response to painful stimuli and the development of chronic headache behaviors^38,39^.

According to the above discussions, increased expression levels of neurotransmitters in the present study were observed in the emotional and behavioral nuclei or regions of rats with MDBC hyperplastic changes. This corresponds to the increased headache-related behaviors in the current study, such as cage scratching, head scratching, and so on. There was strong evidence that chronic headaches could be accompanied by emotional dysfunction such as anxiety and depression. These findings further support the hypothesis that chronic headaches could be induced by hyperplastic changes of the MDBC. Meanwhile, the emotional-related neural conduction pathways in the brain were also activated.

Anatomical and physiological mechanisms have demonstrated that pain originating from various neck structures can refer pain to the head, including the frontal head regions and even the orbit in patients with headaches^40^. The convergence of pain fibers from the trigeminal nerve, including the ophthalmic division of this nerve and from the upper cervical nerves is the anatomical basis for the referred pain from the upper cervical region to the head^41^. Clinical studies have shown that pain caused by cervical structures can refer to the head^42^. Our study coincidentally provides experimental evidence for this mechanism.

There are many pain-sensitive structures in the upper neck. The SDM, as well as other cervical structures dominated by cervical nerves C1-C3, is an extremely pain-sensitive structure^43^. Research shows that atrophy or hypertrophy of the posterior rectus muscles may lead to abnormal levels of traction on the SDM, which may stimulate nociceptive fibers within the SDM^4,8,44,45^. Hack et al.^3^ speculated that the MDB fibers transmit forces from the cervical spine joint complex to the pain-sensitive dura. Based on these findings, hyperplastic changes of the MDBC in our study may disrupt the balance between cervical spine joint complex stability and mobility and stimulate nociceptive fibers within the SDM. These changes may also have triggered the headache.

The central processes of sensory cervical nerves C1-C3 enter the spinal cord at the upper cervical region and converge with neurons of the trigeminal nerve in the trigeminal nucleus caudalis^46^. Nociceptive afferents in the trigeminal nerve include those from the supratentorial dura synapse on the second-order sensory neurons in the trigeminal nucleus caudalis^46^. Both cervical and trigeminal input is transmitted to second-order nociceptive neurons in the trigeminocervical nucleus and then passed along to the thalamus and the cerebral cortex^47^. As was shown by our immunohistochemical staining results, headaches induced by hyperplastic changes of MDBC would provide nociceptive inputs to the second-order neuron of the STN through some potential mechanism, which could then send pain information to the VPM and ACC. But the thalamus and cerebral cortex have no way to distinguish their activation from cervical or trigeminal afferents^41^. That is, the pain induced by the MDBC hyperplasia could be transmitted through the craniofacial pain pathway, and this neurophysiologic pathway might be a referred pain.

However, there are still some limitations. Firstly, hyperalgesia of CGRP and analgesia of 5-HT were simultaneously activated during headache transmission in the present study. The detailed mechanism of this paradoxical result is unknown. Considering the complex regulatory mechanisms of chronic headaches, other neurotransmitters, neuropeptides, and modulators may also be involved. Secondly, it is speculated that abnormal CSF circulation may be the potential mechanism of chronic headaches induced by the pathological MDBC. However, it has not been experimentally demonstrated how the transmission of headache sensation is further activated by abnormal CSF circulation. Thirdly, we only selected bleomycin injection for 4 weeks as the most appropriate observation time, which might not have been long enough to observe the long-term effects of the pathological MDBC on the occurrence and development of chronic headaches. In addition, additional research is also needed to exclude the side effects of bleomycin injection into the posterior atlanto-occipital interspace of rats.

**Figure 4:**
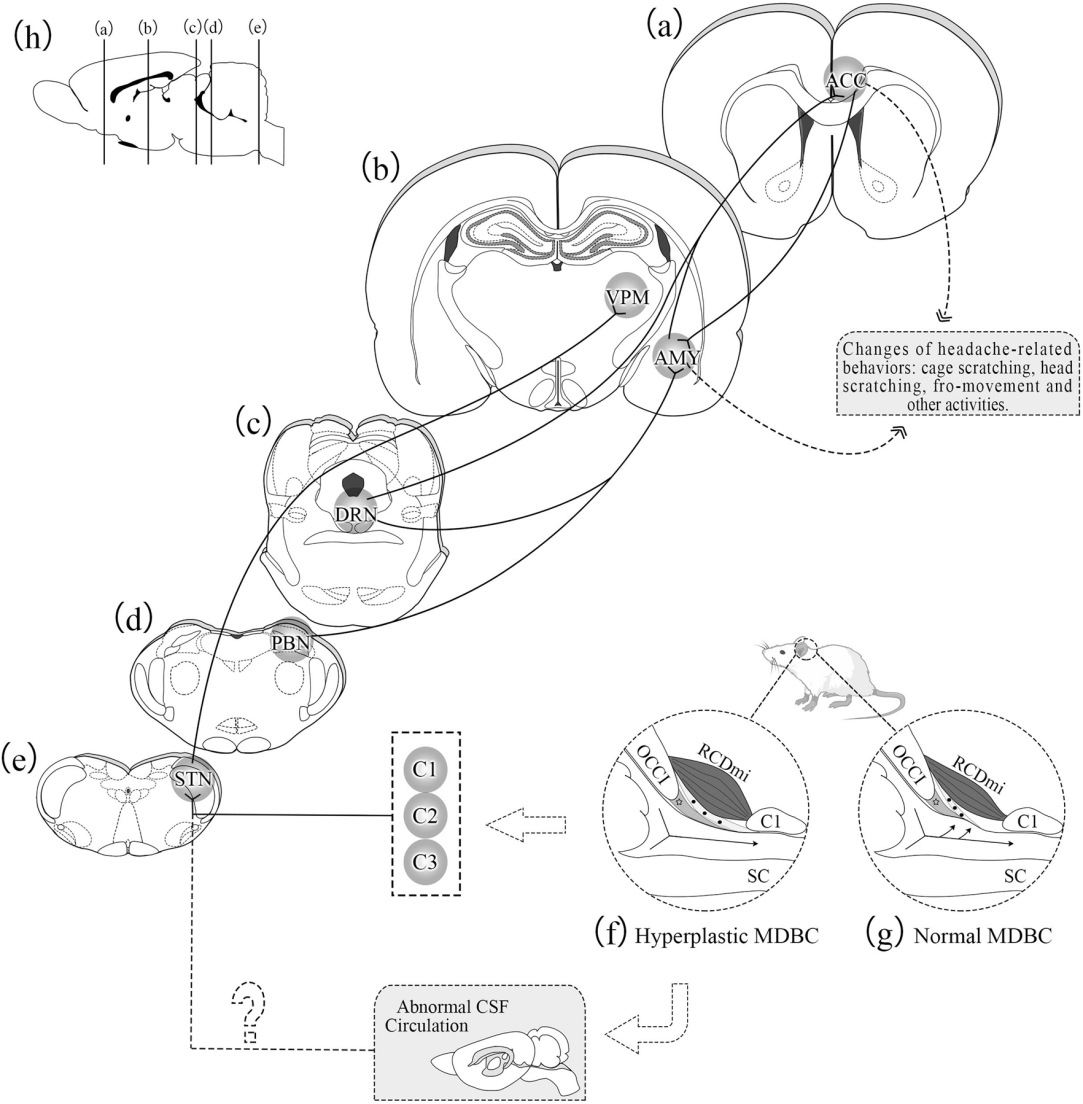
A schematic illustration of a possible underlying mechanism of chronic headaches induced by hyperplastic changes of the MDBC. (f) and (g) are the schematic diagrams of the posterior atlanto-occipital interspace of MDBC hyperplastic rats and normal rats, respectively. When the MDBC (MDB fibers: ● and RCDmi) and the posterior atlanto-occipital membrane (☆) have hyperplastic changes, the flow pattern of CSF ( →) within the cranio-cervical junction can be changed accordingly. The abnormal CSF circulation may activate (dotted line) the conduction of neural pathways in the brain. The nociceptive activity from the spinal dura may then converge on the second-order neuron of the trigeminal nerve and be passed along to the cerebral cortex. (a–e) are the coronal sections of the rat brain where the positive expression nuclei are located. CSF: cerebrospinal fluid; OCCI: occipital bone; C1: atlas; RCDmi: rectus capitis dorsal minor muscle; SC: spinal cord.

In conclusion, the current study provides further support for the view that pathological changes in the MDBC are one of the causes of chronic headaches. As shown in Fig. 4, it is speculated that abnormal CSF circulation or transmission of pain-sensitive SDM induced by abnormal MDBC may be the potential mechanism of some chronic headaches of unknown etiologies. Moreover, evidence for the involvement mechanism of chronic referred pain was also proposed. This study may provide anatomical and physiologic answers to the pathogenesis of some unexplained chronic headaches.

## Materials and methods

### Ethics statement

The study was carried out in compliance with the ARRIVE guidelines. Animals were provided by Dalian Medical University, and experimental protocols involving animals were approved by the Ethics Committee of Dalian Medical University. All the research procedures were carried out in accordance with all relevant guidelines and regulations of Dalian Medical University.

### Animals and models

A total of 18 male 3-month-old Sprague–Dawley (SD) rats (weighing 250–300 g) were included in this study. Briefly, the MDBC hyperplasia model in rats was established as described previously^21^. 18 rats were randomly divided into three groups: bleomycin (BLM) group, PBS group and CTL group (six rats per group). The drug was injected by the microsyringe into the MDBC fibers in the posterior atlanto-occipital interspace of rats under anesthesia. The BLM group received a 25 µl (40 mg/ml) BLM injection on both sides, the PBS group was injected with an equal volume of PBS, and the CTL group was used without any treatment. All surgeries were performed under anesthesia with tribromoethanol, and every effort was made to minimize suffering.

### Behavioral observation

Four weeks after injections, the behaviors of the rats were recorded continuously for 7 days (from 0:00 to 2:00 and from 22:00 to 24:00 every day, as the rats were more active at these two time points) through the monitors hanging above the home cages. Then, the headache-related behavioral motions were collected in the rats of each group, including cage scratching, head scratching, fro-movement, grooming episodes, and nesting^48,49^. One score was given for each behavior. Then summarize the total score of all motions for each rat and use “behavioral score” to represent the headache-related behavior of the rats. All behavioral tests were performed in a double-blinded manner.

### Morphology and morphometry

After the behavioral observation, the rats were euthanized, and the specimens (neck and brain tissues) were removed for fixation. After decalcification of neck tissues, the specimens were routinely processed, embedded in paraffin, and sectioned to a thickness of 8–10 µm. Rat neck tissue specimens in the paracentral median sagittal sections were stained with Masson’s trichrome staining, followed by observation and capturing images under a light microscope. Subsequently, a semi-quantitative analysis of the Masson’s staining images was performed using Image J software (National Institutes of Health, Bethesda, MD). The spinal dura mater, the posterior atlanto-occipital membrane, MDB fibers, and the rectus capitis dorsal minor muscle in the posterior atlanto-occipital interspace were selected as the regions of interest, and the bone areas were excluded. The same area was selected in all sections, and the measurements were limited to the region of interest. The collagen area and total tissue area in the region of interest were measured using Image J. Collagen volume fraction (CVF) was calculated as the ratio of collagen area to total tissue area in this section.

Immunohistochemistry was used to evaluate the calcitonin gene-related peptide (CGRP) and serotonin (5-HT) expression in the brain tissues. After fixation of brain tissues, the specimens were routinely processed, embedded in paraffin, and sectioned to a thickness of 4–6 µm. Anti-CGRP and anti-5-HT (Abcam, USA) were respectively incubated with brain sections after routine dewaxing, hydration, and antigen retrieval. Antibody depositions were visualized using diaminobenzidine (DAB). Nuclei were counterstained with hematoxylin and visualized under a light microscope. The results were analyzed with Image J software. Briefly, the integrated optical density (IOD)/area was calculated and employed as the expression level of CGRP or 5-HT for each nucleus of each rat.

### Statistical analysis

Statistical Package for Social Sciences (SPSS) (version 25.0) was used to analyze the data. GraphPad Prism 8.0 software (GraphPad Software, Inc., La Jolla, CA) was used for making charts. The data was shown as mean ± standard deviation (SD). A one-way analysis of variance (ANOVA) was used for intergroup comparison, and a paired t-test was used for intra-group comparison. A value of P < 0.05 was considered statistically significant.

## Data Availability

All data generated or analysed during this study are included in this article.
